# Green Synthesis of Sodium Alginate/Casein Gel Beads and Applications

**DOI:** 10.3390/nano15060456

**Published:** 2025-03-17

**Authors:** Ruixing Ge, Jiaji Wang, Junlong Piao, Zhenghua Pan, Zhehao Zhang, Yating Yang, Jin Huang, Zhiguo Liu

**Affiliations:** 1 Aulin College, Northeast Forestry University, Harbin 150040, China; geruixing15@nefu.edu.cn (R.G.); 2021214732@nefu.edu.cn (J.W.); michael.benjamin@nefu.edu.cn (J.P.); 2021214727@nefu.edu.cn (Z.P.); easymony_zhang@nefu.edu.cn (Z.Z.); yangyating@nefu.edu.cn (Y.Y.); 2 School of Chemistry, Chemical Engineering and Resource Utilization, Northeast Forestry University, Harbin 150040, China

**Keywords:** sodium alginate, casein, gel, dye removal, adsorption, protein glycosylation

## Abstract

Green-synthesized gel materials can efficiently absorb and remove organic dyes from wastewater. This investigation designed and synthesized a novel modification method of sodium alginate gel beads based on the protein glycosylation reaction (Maillard reaction) using green chemistry principles. The prepared gel beads were subsequently applied to examine their efficacy in adsorbing the organic dye methylene blue. The adsorption process and mechanism were characterized and analyzed. At an adsorption equilibrium of 300 K, the adsorption value can reach 908 mg/g. The dry casein glycosylated gel beads synthesized in this study demonstrate the potential for further development as a novel adsorbent for organic dyes in wastewater.

## 1. Introduction

**Figure 1:**
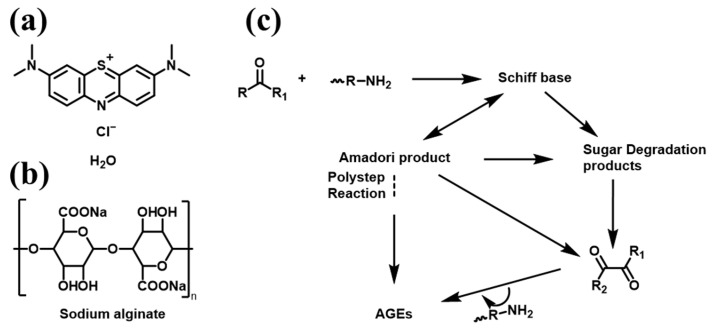
(**a**) Structure diagram of MB; (**b**) SA structure diagram; (**c**) Simplified scheme of the Maillard reaction [17].

Rapid industrial development has led to the advancement of society in numerous aspects. However, industrial wastewater produced by industrial processes can disrupt the ecological equilibrium, contaminate water bodies, alter the ionic balance of water quality, and threaten the health of human beings and other lives. Toxic and recalcitrant dyes discharged into water bodies have reduced the biodiversity of aquatic ecosystems. It can also bioaccumulate through the food chain, potentially endangering human health. Methylene blue (MB) is a widely used dye. When consumed in excess in humans, it can induce adverse symptoms such as dizziness, chest pain, and confusion (Figure 1a). Several wastewater treatment methodologies, including advanced oxidation processes [1], aerobic or anaerobic digestion [2], and coagulation [3], have been used to remove dyestuffs from aqueous solutions. However, these procedures are inefficient, cost-prohibitive, or have detrimental environmental impacts [4]. Consequently, there is a need to develop novel and effective decolorization and decontamination methods. In recent years [5], adsorption methods utilizing biomaterials, mineral oxides, activated carbon, or polymer resins have been proposed. Various unconventional adsorbent materials have been investigated to elucidate their capacity for dye removal [6]. For industrial acceptance, adsorbents must demonstrate efficiency, environmental compatibility, and sufficient availability. Casein conforms to most of these properties and is also utilized as a gel in various domains of adsorption science [7,8].

Casein (CA) accounts for about 80% of the total protein in milk. It is a great source of calcium and phosphorus and contains various essential amino acids for the human body [9]. CA comprises four phosphorylated proteins (*α*-s1-, *α*-s2-, *β*-, and *κ*CNs) that are present in genetically and post-translationally modified variants from bovine sources [10]. As a notable biopolymer, CA is non-toxic and biodegradable. It contains numerous hydrophilic functional groups, exhibits favorable surface activity and stability, and is suitable for preparing hydrogels, rendering it readily obtainable and relatively economical. Sodium alginate (SA) is a widely utilized biomass material that is considered an excellent substrate for preparing adsorbents owing to its high bio-compatibility, biodegradability, and renewability (Figure 1b). It is a linear anionic co-polymer with homopolymeric blocks of (1–4)-linked *β*-D-mannuronate (M) and C-5 epimer *α*-L-guluronate (G) residues covalently linked together in various sequences or blocks. It has been reported that the carboxyl group of SA and the oxygen atom of pyranose can form a stable five-membered chelate with metal ions, thus providing a binding site for the adsorption process [11]. However, the relatively poor properties of SA, such as its stability, mechanical strength, and heat resistance, limit its application in water treatment. Compounding and crosslinking with other materials are two effective methods for enhancing the chemical resistance, mechanical strength, and adsorption capacity of SA-based adsorbents.

The essence of the Maillard reaction is the covalent connection of free amino acid groups in proteins and carbonyl groups of reducing sugars under heating conditions to form Schiff bases (Figure 1c) and further form Amadori compounds [12]. A large number of studies have shown that the solubility, emulsification, and foaming properties of protein-sugar covalent couplings are effectively improved compared with pure proteins [13]. Unlike monosaccharides or disaccharides, the covalent binding of polysaccharides to proteins has a greater advantage in enhancing specific physicochemical properties [14,15,16]. In this study, SA hydrogels were modified for the removal of dyes (MB) from wastewater using the glycosylation reaction (also known as the Maillard reaction) between CA and calcium alginate in CA.

The prepared SA-CA gel beads are called the SA/CA gel beads in this study. SA/CA gel beads were characterized using thermogravimetric analysis (TGA) and SEM electron microscopy. The recycling performance of SA/CA gels was evaluated using a recycling test. The effects of adsorption conditions (temperature, pH of solution, and isoelectric point of binding material) and the material mass ratio (SA:CA) on the adsorption performance of SA/CA gel beads were investigated. The kinetic and thermodynamic aspects of the adsorption process were determined, and the relevant parameters were calculated. Finally, the adsorption mechanism of the SA/CA gel beads was elucidated.

## 2. Materials and Methods

### 2.1. Experimental Reagents

Sodium alginate (SA, biochemical grade) was obtained from Fuchen (Tianjin) Chemical Reagent Co., Ltd. (Tianjin, China). Casein (CA, analytical purity) was supplied by Aladdin Biochemical Technology Co. (Shanghai, China). Sodium hydroxide (analytical purity), methylene blue (MB, analytical purity), and anhydrous calcium chloride (analytical purity) were purchased from Kemio Chemical Reagent Co., Ltd. (Tianjin, China), Tianjin Ruijinte Chemical Co., Ltd. (Tianjin, China), and Tianjin Tianli Chemical Reagent Co., Ltd. (Tianjin, China), respectively. o-Phthalaldehyde (OPA, ≥98% (GC)), β-Mercaptoethanol (≥98% (GC)), and Sodium tetraborate decahydrate (Decahydrate, reagent grade, 99.5%) from Shandong Keyuan Biochemical Co., Ltd. (Shandong, China). Sodium dodecyl sulfate (SDS, reagent grade, 92.5–100.5%) is produced by Shanghai Macklin Biochemical Technology Co., Ltd. (Shanghai, China). All reagents were used without further purification.

### 2.2. Preparation of SA/CA Samples

To prepare the 2 wt.% CA solution, 1 g of casein, and 50 mL were added to the beaker, then the pH was adjusted to 12 with 1 M of NaOH solution. The solution was stirred at 50 °C for 30 min until it was completely dissolved.

Then, 50 mL of the above solution was taken, and 1.0 g of SA was added (1:1). The mixture was stirred for 30 min until it was fully dissolved and no bubbles remained (it took about 18 h to be free of bubbles). The solution was placed into a 100-mL round-bottomed flask. It was heated in a 50 W microwave for 20 min, cooled to room temperature, and then 0.5 g of SA was added to complete the gel beads’ shape. Another 20 mL of 5 wt.% CaCl_2_ solution was taken in a beaker and added with a peristaltic pump at a rate of 0.2 mL per drop. After 6 h of cross-linking, wash with ultra-pure water three times. Prior to the characterization of SA/CA gel beads (SEM, TGA, and FT-IR), we performed a freeze-drying process. SA/CA hydrogel beads were freeze-dried for 90 h (freezing the first cycle from −65 °C, lifting 5 °C every 5 h until the end of 20 °C, maintaining a complete cycle of 20 °C to 90 h) in the freeze dryer (TF-FD-1, SCIENTZ Biotechnology, Zhejiang, China).

### 2.3. Morphological Analysis by Scanning Electron Microscopy (SEM)

The SA/CA samples were also freeze-dried before analysis. The morphology of the SA/CA gel beads was characterized using a scanning electron microscope (lower multiple: JSM-7500F; higher multiple: Regulus8100). For this study, surface and cross-sectional samples of pure SA gel beads, SA/CA gel beads, and SA/CA gel beads after 24 h of adsorption were prepared for SEM analysis. To enhance the conductivity of the samples, the gel beads were coated with platinum using a metal sputter coater.

### 2.4. TGA Analysis

The SA/CA samples were also freeze-dried before analysis. The thermal stability measurements were performed at (N_2_) atmosphere with a heating rate of 10 °C/min from room temperature to 600 °C using a simultaneous thermal analyzer (HITACHI STA200).

### 2.5. Adsorption-Desorption Cycle Test

After the adsorption, the adsorbed SA/CA gel beads were put into a 0.1 M HCl solution for desorption for 2 h. The resolved SA/CA gel beads were cleaned with deionized water several times, and the adsorption experiment was carried out in the next cycle. The adsorption value and removal rate were recorded in each cycle.

### 2.6. Maillard Reaction Verification and Definition of Protein and Alginate Content in Materials

Preparation of OPA reagent: Weigh 40 mg of OPA and dissolve it in 1 mL of methanol. Add 2.5 mL of 20% SDS solution, 0.1 M borax solution to 25 mL, and 100 μL β-mercaptoethanol, and set the volume to 50 mL.

Sample determination: Absorb 200 μL of glycosylation graft product solution, add 4 mL of the prepared reagent into a test tube, mix well, and react in a water bath at 35 °C for 2 min. The absorbance value A_1_ is measured at 340 nm. Under the same conditions, the absorbance value A_0_ of the 200 μL pre-reaction solution is the blank sample, and the difference ΔA is the net absorbance value of the free amino group. The decrease in free amino acid content before and after the reaction was determined by OPA to indicate the occurrence of glycosylation [18].

$$DG=\frac{\left(A_{0}-A_{1}\right)}{A_{0}}\times 100\%$$

The browning degree is determined by diluting the sample solution (1 mg/mL) 25 times with SDS solution (0.1%, *w*/*w*) and measuring the absorbance at 420 nm. The browning degree is expressed as A_420_ [19].

### 2.7. Adsorption Performance Study

#### 2.7.1. MB Standard Curve

**Figure 2:**
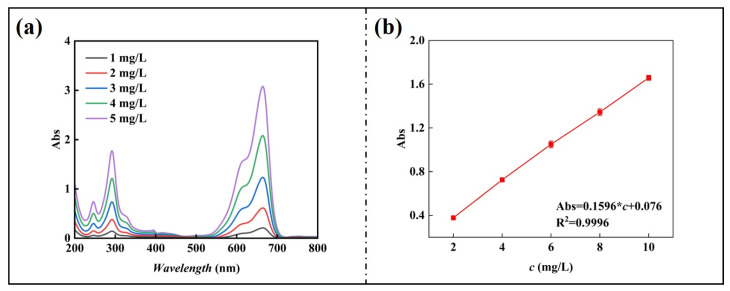
(**a**) MB absorption spectrum (665 nm); (**b**) standard equation curve of MB.

First, 0.5 g of pure MB was weighed and dissolved in a small amount of water in a beaker. The solution was transferred to a 500-mL dark-colored volumetric flask and shaken well to prepare a 1000 mg/L MB solution. The mother liquor was diluted to 100 mg/L and further diluted to 5 mg/L, 4 mg/L, 3 mg/L, 2 mg/L, and 1 mg/L. A UV–Vis measurement (Persee TU-1901) was used to detect the different concentrations of MB, and the standard solution was obtained. The UV absorption spectra of MB were obtained, as shown in Figure 2a.

The maximum absorption wavelength of the MB solution was 664.2 nm, and the absorbance at 665 nm was used to plot the standard curve of concentration versus absorbance (c_0_ = 10 mg/L, 8 mg/L, 6 mg/L, 4 mg/L, and 2 mg/L), and the results are shown in Figure 2b [20,21].

#### 2.7.2. MB Adsorption Test

A systematic adsorption experiment was carried out using MB as a typical pollutant to investigate the adsorption capacity of SA/CA hydrogels as a kind of adsorbent material for dyes. Adsorption experiments were performed at 300 K (27 °C) to simulate near-room temperature conditions (25 °C) and close to the number to achieve visual neatness, which is relevant for potential biomedical or environmental applications. The SA/CA hydrogels (0.5 g/L) were added into a beaker containing MB solution (300 mg/L, 50 mL) as adsorbent and adsorbed at 300 K for 1440 min with an oscillation rate of 120 r/min. After adsorption, the supernatant in the beaker was taken, and the absorbance of MB before and after the adsorption was determined by a UV spectrophotometer so that the adsorption value was calculated. The supernatant was diluted appropriately for MB solutions with initial concentrations exceeding 10 mg/L to ensure absorbance values fell within the calibrated range (0–10 mg/L).

Isoelectric point: To prepare a sodium chloride solution, 0.1 M HCl solution and 0.1 M NaOH solution were used to adjust the pH value of the solution. A series of sodium chloride solutions with a concentration of 0.01 M between pH = 2 and pH = 12 were obtained. Then, 10 mL of sodium chloride solution of different pH was taken, and 15 mg of gel materials was added into the iodine bottle and oscillated continuously at 300 K for 1440 min. The pH of the solution before oscillation is denoted pHi, and the pH of the solution after oscillation is denoted pH_f_. pH_i_ is the horizontal coordinate, ΔpH (pH_i_-pH_f_) is the vertical coordinate, and the intersection of ΔpH and pH_i_ is denoted as the isoelectric point of the gel materials [22].

The effects of different SA/CA hydrogel samples on the adsorption performance of MB were determined by the adsorption experiments with different SA/CA hydrogels. The effects of the solution’s initial pH on the adsorption performance were determined by varying the initial pH 2–11, which was also adjusted by HCl and NaOH.

### 2.8. Theoretical Background and Data Analysis

In adsorption experiments, the adsorption value q_e_ (Equation (1)) and q_t_ (Equation (2)) are two important parameters to measure the adsorption capacity. The adsorption value is the mass of pollutant adsorbed per unit mass of adsorbent. q_e_ represents the adsorption value when the adsorption equilibrium is reached, and q_t_ represents the adsorption value at time t. The values of adsorption values q_e_ (mg/g) and q_t_ (mg/g) can be calculated by the following equation [23].

(1) $$q_{e}=\frac{(c_{0}-c_{e})\times V}{m}$$

(2) $$q_{t}=\frac{{(c}_{t}-c_{e})\times V}{m}$$

where *c_t_* (mg/L) and *c*_0_ (mg/L) correspond to the concentration of MB at time *t* in the solution and initial MB concentration, *c_e_* (mg/L) is the concentration at which adsorption reaches equilibrium. The *m* (g) is the weight of the added SA/CA gel beads, and *V* (L) represents the volume of the solution [24].

Adsorption kinetics is an indispensable analytical tool to analyze the adsorption process. Kinetic models usually assist the use of adsorption kinetics to examine the adsorption process. The effects of adsorption time on the adsorption performance were investigated by varying the adsorption time of oscillation (0–1440 min). The kinetic analysis included the application of a linear intra-particle diffusion model (Equation (3)) [25] and nonlinear models: pseudo-first-order (Equation (4)) [26] and pseudo-second-order (Equation (5)) [27], to which the following equations corresponded:

(3) $$q_{t}=K_{id}t^{\frac{1}{2}}+C$$

(4) $$q_{t}=q_{e}(1-e^{-k_{1}t})$$

(5) $$q_{t}=\frac{k_{2}q_{e}^{2}t}{1+k_{2}q_{e}t}$$

where *K_id_* (mg/g·min^1/2^) corresponds to the internal diffusion coefficient, *C* (mg/g) is the boundary layer’s thickness, *k_1_* (min^−1^) and *k_2_* (g/mg·min) are the first- and the second-order sorption rate [24].

Adsorption isotherm is an important tool to study the interaction between adsorbent and adsorbate. The effects of temperature and initial concentration on the adsorption performance were determined by varying the initial concentration (50–1000 mg/L). The functional relationship between the amount of the sorbed material on the sorbent and its concentration in the solution in contact with the sorbent is expressed by the sorption isotherms. Isotherm models employed for analyzing equilibrium data were Langmuir (Equation (6)) [28], Freundlich (Equation (7)) [29], Sips (Equation (8)) [30], and Temkin (Equation (9)) [31]:

(6) $$q_{e}=q_{m}\frac{K_{L}c_{e}}{1+K_{L}c_{e}}$$

(7) qe=KFce1n

(8) $$q_{e}=\frac{q_{m}{\left(\beta_{S}c_{e}\right)}^{n}}{1+{\left(\beta_{S}c_{e}\right)}^{n}}$$

(9) $$q_{e}=\frac{RT}{b}ln(K_{T}c_{e})$$

whereby maximum capacity of sorption corresponds to *q_m_* (mg/g), respectively, Langmuir constant of sorption energy corresponds to *K_L_* (L/mg), Freundlich constant about the capacity of sorption is denoted by *K_F_* (mg/g)·(L/mg)^1/n^, exponent related to the sorption intensity is represented by *n*, Sips constant associated with sorption affinity is denoted by *β_S_* (L/mg), the universal gas constant is denoted by *R* (8.314 J/mol·K), and absolute temperature is denoted by *T* (K). In contrast, *K_T_* (L/g) and *b* represent Temkin constants.

Thermodynamic parameters were determined through MB sorption experiments carried out at 290, 300, and 310 K employing the subsequent formulas [32]:

(10) $$\Delta G^{0}=-RTlnK_{D}$$

(11) $$K_{D}=\frac{c_{a}}{c_{e}}$$

(12) $${lnK}_{D}=\frac{\Delta S^{0}}{R}+\frac{\Delta H^{0}}{RT}$$

(13) $$\Delta G^{0}=\Delta H^{0}-T\Delta S^{0}$$

whereby ∆*H*^0^, ∆*S*^0^, and ∆*G*^0^ stand for enthalpy change, entropy change, and Gibbs free energy change, respectively. *K_D_* represents the constant of linear sorption distribution, and *c_a_* (mg/L) and *c_e_* (mg/L) correspond to the concentration of sorbed MB and the concentration of MB remaining in the solution, respectively. Equation (13) expresses the correlation between temperature, the equilibrium constant *K_D_*_,_ and the change in Gibbs free energy.

Origin 2021 software (OriginLab Corporation, Northampton, MA, USA) was used to fit and plot the obtained data. According to this software, the standard error is obtained and reflected in the main data in the article. Additionally, to calculate mean relative deviation (*MRD*) using this equation [33]:

(14) $$MRD=\frac{\sum_{i=1}^{i=n}\left|q_{t,exp}-q_{torm,cal}\right|}{\bar{q_{e,exp}}*n}*100$$

### 2.9. Infrared Analysis by Fourier Transform Infrared Spectroscopy (FT-IR)

The SA/CA samples were freeze-dried and prepared by weighing at a ratio of KBr to a sample of 100:1. The mixture was then ground into a fine powder in a clean agate mortar for 10 min under fluorescent light. The powdered mixture was placed into a mold and pressed into a pellet for 30 s to 1 min. The pellet was then removed and mounted on a sample holder. After background subtraction, the samples were analyzed using an FTIR spectrophotometer (Thermo Nicolet-IS10). This experiment primarily examined three types of samples: pure SA gel beads, SA/CA gel beads, and SA/CA gel beads after 24 h of adsorption. The aim was to compare and verify the success of the Maillard reaction loading and to explore the primary possible adsorption mechanisms.

## 3. Results

### 3.1. SEM Analysis

**Figure 3:**
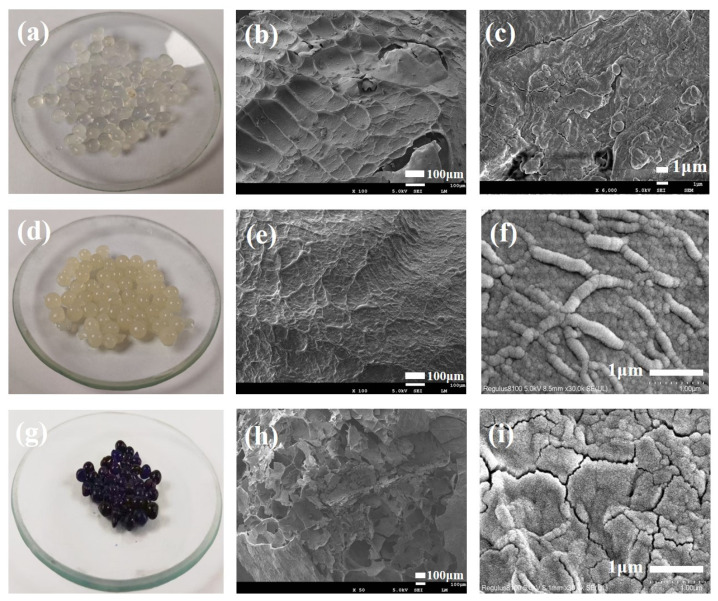
SEM and digital pictures of pure SA gel beads (**a**–**c**), SEM and digital pictures of SA/CA gel beads (**d**–**f**), and SEM and digital pictures of SA/CA gel beads after adsorption of MB (**g**–**i**).

The surface morphology of gel beads before and after loading CA is shown in Figure 3a–f. Two different kinds of gel beads are both uniform and spherical; the SA gel beads are white, transparent gel beads. The transparency of the gel beads decreases with the addition of CA, but the color remains unchanged. The diameter of the two different gel beads is about 3 mm, as shown in Figure 3a,d. At high magnification, the surface of SA/CA gel beads became rough due to the addition of CA. At the same time, loading of CA makes gel beads avoid cracking caused by freeze-drying. Compared with SA gel beads, a number of particles with a diameter of less than 1 micron were precipitated on the surface of SA/CA gel beads (Figure 3f), and these nanoscale substances may be precipitated protein aggregates/grafted products, which may be the main reason why SA/CA gel beads have stronger capacity than ordinary SA gel beads. It indicates that CA was successfully introduced into the gel materials, as shown in Figure 3b,c,e,f [34].

The digital photos and SEM images of the interface cross-section before and after SA/CA gel beads adsorbing MB are shown in Figure 3g–i. After adsorption, the gel beads changed from yellow and white to black and purple (Figure 3d,g). It can be seen from the SEM image of the cross-section that the SA/CA gel beads show a three-dimensional porous structure before adsorption, and such a structure is still preserved after adsorption (Figure 3h), which proves that this material may have excellent performance in repeated use (adsorption-desorption cycle) [35,36,37]. In the high-magnification image, the round particles on the surface of SA/CA gel beads can be seen as the more realistic morphology of the precipitated proteins. However, the adsorption of MB destroys the surface structure of the original SA/CA gel beads, making the protein precipitation less obvious under the high-power SEM image (Figure 3i), and the possible decrease in gel strength also leads to the cracking of the gel after lyophilization.

### 3.2. TGA Analysis

**Figure 4:**
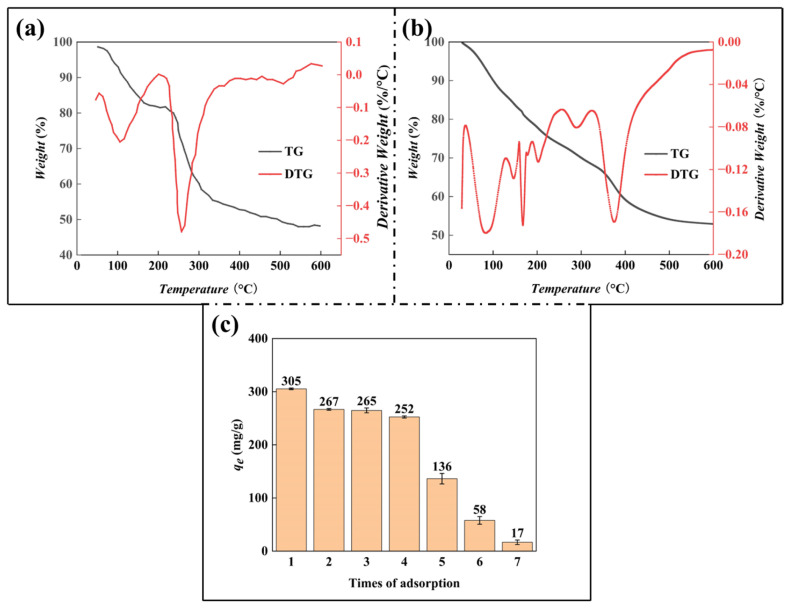
(**a**) TG and DTG curves of SA gel beads; (**b**) TG and DTG curves of SA/CA gel beads; (**c**) Reusability of SA/CA gel beads for MB adsorption. (0.5 g/L SA/CA beads, 300 mg/L, and 50 mL MB, pH = 7.0, T = 300 K).

In order to determine the effect of the CA addition on the thermal performance of gel beads, thermogravimetric tests were conducted on the freeze-dried SA gel beads and SA/CA gel beads. The test results are shown in Figure 4a,b. Both SA gel beads and SA/CA gel beads exhibit two main stages of weightlessness. The first identical weight loss stage occurs at 30–200 °C, and this part of the mass loss is attributed to the volatilization of free and adsorbed water in the sample. However, SA/CA gel beads exhibit more small stages of weightlessness at this stage, mainly because the loss of crystal water caused by different types of CA is quite different from that of SA. The second weight-loss stage occurs at 200–500 °C, and the mass loss in this part is mainly caused by thermal decomposition and carbonization of biomass materials [38]. However, compared with SA gel beads, SA/CA gel beads have more weight loss periods during the period from 150 °C to 400 °C, mainly because the introduction of CA increases the organic content of unit gel. The modification process of the Maillard reaction increases the content of chemical bonds (ester bonds), thus adding several different weight-loss stages in the figure.

In addition, the maximum decomposition temperatures of SA and SA/CA gel beads are 251 °C and 375 °C, respectively. SA/CA gel beads show higher maximum decomposition temperatures (the weight loss platform of SA gel beads is between 150 and 250 °C, and the weight loss platform of SA/CA gel beads is around 350 °C), which is attributed to the hydrogen bond between CA and SA molecular chains. In addition, the residual carbon content of SA gel beads and SA/CA gel beads at 600 °C is different, 48.6% and 52.93%, respectively. In contrast, the residual carbon content of SA/CA gel beads is higher, indicating that SA/CA gel beads have strong thermal stability. Significantly, when the temperature rises to 350 °C, further decomposition reactions in SA/CA gel beads produce more gaseous products, increasing weight loss. In fact, the experimental conditions of this experiment were mostly lower than 100 degrees (aqueous solution environment), and the main purpose of thermogravimetry was to demonstrate the successful grafting of CA and partially analyze its thermal decomposition properties, and temperatures that were too high for thermogravimetry were of little significance.

### 3.3. Adsorption-Desorption Cycle Test

Figure 4c is the result of the adsorption and regeneration experiment of MB by SA/CA gel beads. After six cycles of adsorption and regeneration, the adsorption capacity decreased significantly. The possible reason for the decrease in adsorption capacity was the slight weight loss of SA/CA gel beads and the decrease in adsorption sites during the regeneration process. The decrease was evident at the beginning of the first cycle (about 12.54%). In the following second and third cycles, the decrease was smaller, 0.76% and 4.64%, respectively, but the adsorption capacity remained above 250 mg/g. In the fourth cycle, it dropped to about 136 mg/g, a decrease of about 46%. It even dropped to 16.7 mg/g on the second two passes (the seventh adsorption). The reason for this characteristic, we predict, is the decline in the stability of the material. This experiment proves the successful reuse of SA/CA gel beads.

### 3.4. Maillard Reaction Verification

After experimental verification, the degree of graft (*DG*%) of SA/CA gel beads is about 10–15% (Table 1). To increase the molding performance of the hydrogels, we added 50% SA (resulting in the actual mass ratio of SA to CA of 1.5:1), so this experiment will use 1 mg/mL CA and 1 mg/mL SA as references to prepare reference solutions to judge the degree of graft and the smooth progress of the Maillard reaction. Browning degree is often used to indicate the occurrence of Maillard reactions in foods because it is easier to measure than a degree of graft. It also means that the Maillard reaction has an advanced stage [39], and its specific parameters are shown in Table 1.

The data obtained from these indirect measurements may contain significant errors and are only of a qualitative nature. The grafting of biopolymers can also be confirmed by using infrared spectroscopy.

### 3.5. Effect of Different Samples on the Adsorption Effect and the Effect of the Initial pH of the Solution on the Adsorption Effect

**Figure 5:**
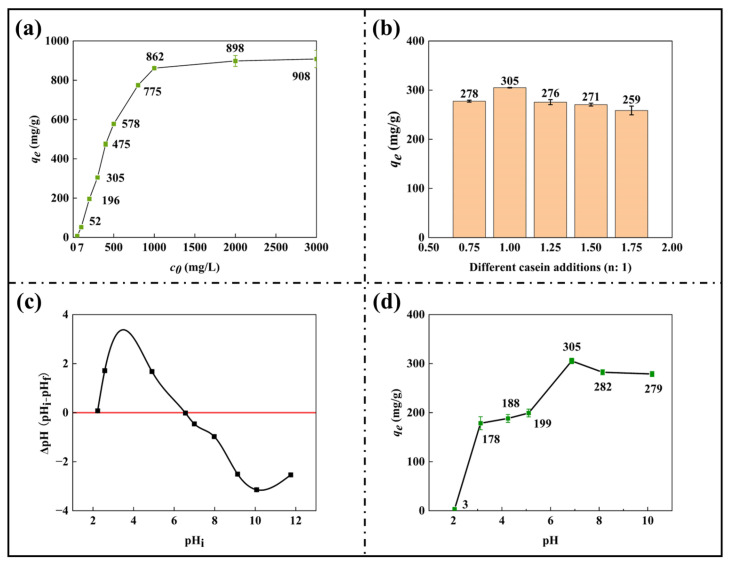
(**a**) Changes of *q_e_* of SA/CA gel beads under different *c*_0_; (**b**) Adsorption capacity of SA/CA to MB at different MB concentrations; (**c**) Changes in *q_e_* at different CA concentrations (n:1); (**c**) pH_pzc_ of adsorbent used in the study; (**d**) Adsorption capacity of SA/CA to MB at different pH values (0.5 g/L SA/CA beads, different concentrations, and 50 mL MB, different pH values, T = 300 K).

The ion exchange reaction between sodium alginate and calcium chloride formed a stable “Egg box” structure [40], and the calcium in the gel was in the form of calcium alginate. For the concentration of adsorbent MB, the error caused by dilution becomes significant when the concentration exceeds 3000 mg/L, leading to inaccurate analysis. Still, the *q_e_* when it reaches equilibrium is about 908 mg/g (Figure 5a). This represents the maximum adsorption capacity of SA/CA gel beads and is also the original data for the adsorption thermodynamic analysis below. In Figure 5a, the values of initial concentration *c* are successively 50, 100, 200, 300, 400, 500, 800, 1000, 2000, and 3000 mg/L. Under the same conditions, the adsorption properties of SA/CA hydrogels with different CA additions (1.75:1, 1.5:1, 1.25:1, 1:1, and 0.75:1) on MB were studied (Table 2), and the results are shown in Figure 5b. Under the initial MB concentration of 1000 mg/L, the reported *q_e_* of SA gel beads in the literature was 800 mg/g [41]. Our experimental results showed a *q_e_* value of 805 mg/g at the same initial concentration, which is consistent with the literature value. However, considering the potential errors introduced during the dilution process of high-concentration solutions and from environmental protection and economical wastewater treatment perspectives, we selected 300 mg/L as the optimal testing concentration. Under this optimized condition, the adsorption capacity of SA hydrogels for MB was determined to be 279 mg/g. It can be seen from the figure that when the mass ratio of SA and CA is 1:1, the adsorption capacity of SA/CA hydrogels increases to 305 mg/g. When the mass ratio is 0.75:1, the product’s adsorption capacity decreases due to insufficient grafting in the reaction. Similarly, the adsorption capacity of SA/CA will also decrease with the addition of CA because the excessive addition of CA will prevent the emergence of adsorption sites, while the excessive addition of reactants will lead to unnecessary waste. Therefore, this paper will choose 1:1 as the ratio of SA/CA preparation. At the same time, the actual microwave catalysis decreased the binding ability due to the participation of some polysaccharides in the reaction, and the original concentration could not drop into a ball smoothly, so part of 0.5 g SA solid was added after grafting to dissolve the gel beads to achieve integrity.

**Figure 6:**
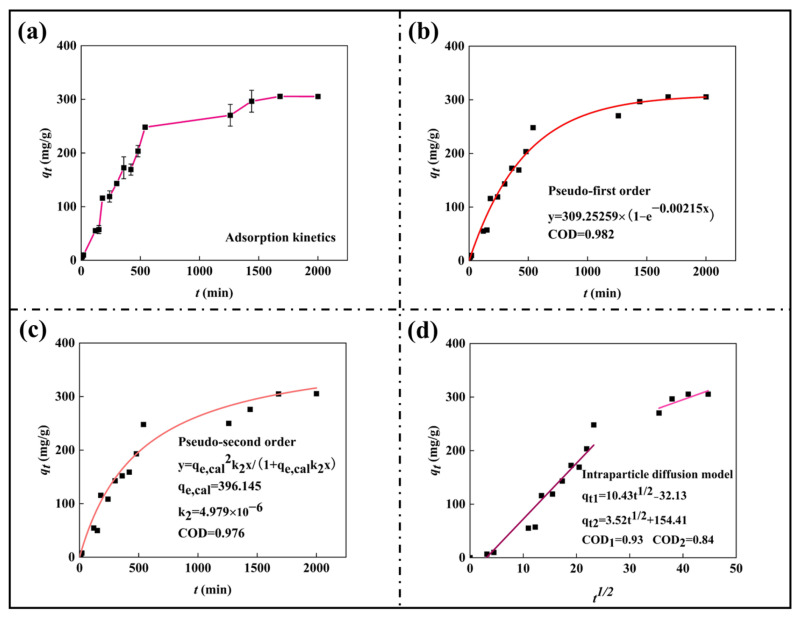
(**a**) Influence of contact time on adsorption of MB by SA/CA (raw data); (**b**) Pseudo-first-order curve of MB adsorption by SA/CA; (**c**) Pseudo-second-order curve of MB adsorption by SA/CA; (**d**) Linear intra-particle diffusion curve of MB adsorption by SA/CA (0.5 g/L SA/CA beads, 300 mg/L and 50 mL MB, pH = 7.0, T = 300 K).

The pH_pzc_ represents the pH value at which the surface of the material is neutral. The isoelectric point for gel beads obtained by plotting ∆pH against pH_i_ is shown in Figure 6c [42]. This is a very important parameter in the adsorption process and gives information about the pH range in which cationic or anionic dye adsorption is feasible. At pH < pH_pzc_, the adsorption of anionic dyes is more feasible because of the positively charged surface of the adsorbent. However, at pH ˃ pH_pzc_, the cationic dye adsorption is more viable because of the negative group on the surface [22,43]. SA/CA gel beads show pH_pzc_ around 6.55; at this point, the surface charge of the beads is approximately neutral. When examining the adsorption parameters of MB, we included conditions with different initial pH values to assess the effect of pH on the adsorption reaction. As shown in Figure 5d, when the pH is less than the isoelectric point of 6.55 of the material, the adsorption efficiency decreases with the decrease in pH due to the positive charge on the surface of the gel beads and the competitive adsorption between hydrogen ions and MB. However, when the solution pH is greater than the pH_pzc_ of the material, the negative charge will facilitate the adsorption of MB, which is reflected in Figure 5d; when the pH is greater than or equal to pH_pzc_, the gel presents a relatively good *q_e_* accompanied by small fluctuations.

### 3.6. Adsorption Kinetics Studies

To better describe the adsorption process, the mechanism of SA/CA hydrogels in MB adsorption was analyzed, and the experimental results were analyzed using kinetic models. The fitting results are shown in Figure 6a–c, and the fitting parameters are shown in Table 3. The pseudo-first kinetic model has a good fitting effect on the adsorption process, and the *r*^2^ of the model is 0.982. In addition, the adsorption capacity predicted by the pseudo-first-order kinetic model is similar to the measured results, indicating that the pseudo-first-order kinetic model has good applicability to the adsorption of MB on SA/CA hydrogels, and the adsorption process is mainly physical adsorption [44]. However, in the fitting process of the pseudo-second kinetics, it can be seen that even though the fitting of the pseudo-second kinetics is relatively poor (85.26%), if we take 400 min as the limit of segmentation, we can see that the fitting before 400 min is poor, but after 400 min, the fitting is quite good. In the adsorption process of MB, SA/CA is mainly physical adsorption. Still, after 400 min, it is both physical adsorption and chemisorption, which is also consistent with the gradual increase in slope in the first stage of the internal diffusion model (although it is the first stage)—the addition of chemisorption leads to an increase in adsorption rate [45].

Due to the difficulty of the above kinetic model in describing the diffusion process of MB during adsorption, the in-particle diffusion model was fitted to the experimental data, and the fitting results are shown in Figure 6d. The fitting results indicate that the adsorption process was carried out in stages. The fastest adsorption is in the first stage; MB spreads rapidly on the surface of SA/CA and occupies the adsorption site on the surface. The second stage of adsorption slows down, indicating that the adsorption site of the surface layer is occupied by MB molecules arriving first, and the MB molecules arriving later diffuse to occupy the internal active sites (hydrogen bonds, etc.). In the preliminary desorption experiment, the gel beads with complete desorption still appear blue, which can prove this problem from the side. The analysis results do not pass through the origin of the coordinates, which indicates that diffusion within the particle is not the only factor limiting the adsorption rate and that the adsorption rate is also affected by other adsorption factors [46,47].

### 3.7. Adsorption Isotherm Studies and Adsorption Thermodynamics Study

**Figure 7:**
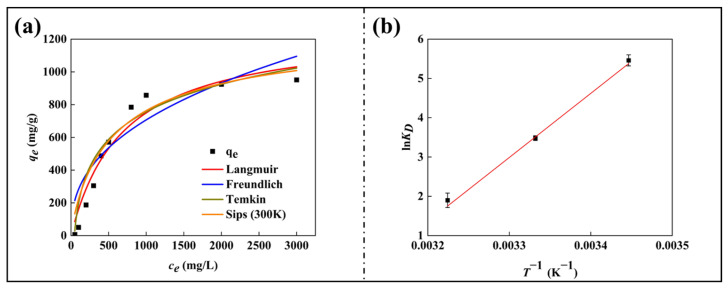
(**a**) Graphical representation of *q_e_* vs. ce for applied isotherm models; (**b**) The linear thermodynamic plot of lnKD vs. *T*^−1^ (0.5 g/L SA/CA beads, different concentrations and 50 mL MB, pH = 7.0, T = 290/300/310 K).

The experimental adsorption data were analyzed using the Freundlich, Sips, Langmuir, and Temkin isotherm models to elucidate potential adsorption mechanisms (Figure 7a). The Sips model exhibited the highest correlation coefficient (*r^2^* = 0.99) and a moderate *MRD* (18.48); its dimensionless heterogeneity parameter (*n* = 0.85) was close to 1, indicating a transition to the single-layer adsorption behavior characteristic of the Langmuir model rather than heterogenous Freundlich-type adsorption [48]. This observation is consistent with the *MRD* data of the Langmuir model being lower than that of the Freundlich model, which further confirms the applicability of the Langmuir model to describe the adsorption process. The Langmuir model (*r*^2^ = 0.95) implies dominant monolayer adsorption at a binding site with uniform energy, although residual surface heterogeneity may cause small deviations. The Freundlich model (*n* = 2.21, *r*^2^ = 0.97) shows moderate consistency, which is consistent with the theoretical framework of multilayer adsorption on heterogeneous surfaces. The Temkin model (*r*^2^ = 0.97) was used for complementary analysis, and the adsorption heat parameter *b* = 10.27 and the equilibrium constant *Kt* = 0.023 were obtained, indicating the existence of an exothermic process (Table 4). The analysis of the Sips, Langmuir, and Freundlich models showed that SA/CA gel beads dominated monolayer adsorption at homogeneous sites in the MB adsorption process, and there may be a small amount of residual heterogeneity. The whole adsorption process is an exothermic interaction [49,50].

The effect of temperature (290 K, 300 K, and 310 K) on the adsorption affinity of SA/CA gel beads was investigated while other optimal parameters were kept fixed. The calculation results of the thermodynamic parameters ∆H^0^, ∆S^0^, and ∆G^0^ (three different temperatures) are shown in Table 5. Using the Van’t Hoff graph of the dependency of lnK_D_ on T^−1^, ∆S^0^ was calculated from the diagram’s intercept and ∆H^0^ from the slope (Figure 7b). The slight decrease in q_m_ value with increasing temperature indicates that the adsorption process is exothermic. And the adsorption efficiency is higher at low temperatures. Therefore, when the temperature rises, the adsorption force of SA/CA gel beads on MB decreases. Negative values of ∆G^0^ obtained at all temperatures confirm that the adsorption process occurs spontaneously at lower temperatures. Based on the enthalpy value of −135.63 kJ/mol, adsorption is exothermic and can be identified as the emergence of chemisorption. The resulting negative entropy indicates that the degree of confusion is reduced, and the alignment is good at the boundary of the adsorbent-SA/CA gel beads phase.

### 3.8. Mechanism Analysis

**Figure 8:**
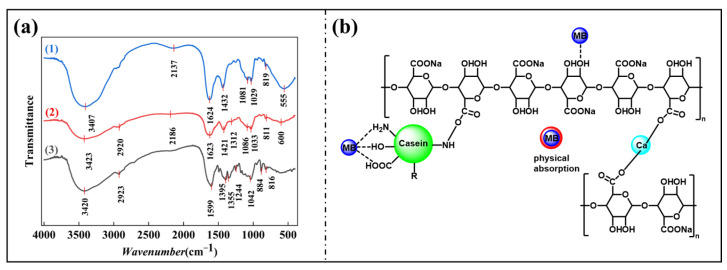
(**a**) FT-IR spectra of SA (1) and FT-IR spectra of SA/CA before (2) and after (3) adsorption of MB, (**b**) Possible mechanism of adsorption of MB by SA/CA gel beads.

Curve (1) in Figure 8a is the infrared spectral curve of pure SA gel beads. Its peak at 3407 cm^−1^ is the stretching vibration of -OH, and its peak at 1624 cm^−1^ corresponds to the stretching vibration peak of C=O. The peaks of 1431 cm^−1^ correspond to the bending vibrations of -OH and -CH, while the 1080 and 1029 cm−1 peaks correspond to the stretching vibrations of C-O and -C-O-C-. The peak of SA/CA (curve (2)) at 3243 cm^−1^ is still the -OH tensile vibration, and the peak at 2919 cm^−1^ is from the -CH tensile vibration of -CH_2_ and -CH_3_ groups, which is not obvious in curve (1), and the corresponding peak is the emergence/enhancement of the newly added -CH_2_- group after CA loading. In contrast, curve (1) does not because the peak of the cycloalkanes is shifted towards the large -OH peak of 3407 cm^−1^. The above analysis and the validation of the Maillard reaction in Section 3.5 demonstrate the success of grafting. Curve (3) is the infrared spectrum absorption peak curve after the SA/CA adsorption of MB. It can be seen that a new peak appears at 883 cm^−1^, which is explained by the vibration of C-S=C, a unique structure in MB. Due to the limitations of experimental conditions, this experiment could not effectively distinguish the peak of -OH from the peak of the hydrogen bond. Still, according to the polarity of the two and the presence of N atoms in MB, it was reasonable to guess that there was a hydrogen bond adsorption process in the adsorption process (Figure 8b).

### 3.9. Comparison, Advantages, and Limitations

The primary advantages of the SA/CA gel beads include the following:

(1) High adsorption capacity (908 mg/g at 300 K), surpassing many reported biomass-based adsorbents (Table 6);
(2) Utilization of green chemistry principles, avoiding toxic cross-linking agents;
(3) Excellent biodegradability and renewability due to the natural origins of SA and CA.

However, this study has certain limitations, such as the following:

(1) The adsorption performance was only evaluated for MB; generalization to other dyes requires further validation;
(2) The long-term stability and reusability of the gel beads in practical wastewater treatment scenarios were not thoroughly investigated;
(3) The scalability of the synthesis process for industrial applications remains to be explored.

**Table 6:** The adsorption parameters of this experiment are compared with those of previous studies (0.5 g/L SA/CA beads, different concentrations, and 50 mL MB, pH = 7.0, T = 300 K).

Absorbent Materials	*q_e_*(mg/g)	Adsorption Reaction Condition	References
Magadiite-chitosan composite beads	45	303 K, *c*_0_ = 100 mg/L, 40 mL	[51]
Alg-g-AO	24	303 K, *c*_0_ = 50 mg/L, 25 mL	[52]
Carboxymethyl cellulose/k-carrageenan/montmorillonite beads	12	303 K, *c*_0_ = 100 mg/L, 50 mL	[53]
CGC/SA	387	298 K, c_0_ = 400 mg/L	[54]
m-ALG/PESA gel beads	400	293 K, *c*_0_ = 500 mg/L, 25 mL	[55]
PVA/SA/Fe3O4@KHA gel beads	782	293 K, *c*_0_ = 1000 mg/L, 50 mL	[56]
SA/CA gel beads	300 K, 50 mL, *c*_0_ = 50 mg/L, *q_e_* = 52 mg/g *c*_0_ = 500 mg/L, *q_e_* = 474 mg/g *c*_0_ = 1000 mg/L, *q_e_* = 862 mg/g		This work

## 4. Conclusions

The SA/CA hydrogels synthesized through ionic cross-linking of sodium alginate (SA) and casein (CA) with CaCl₂ demonstrated efficient methylene blue (MB) adsorption capacity (experimental adsorption equilibrium: 908 mg/g at 300 K). With the support of SEM analysis, a series of tests proved that compared with pure SA gel beads, CA incorporation (SA:CA mass ratio = 1:1) improved the adsorption performance. FT-IR spectra and Maillard reaction indices (*DG*% = 14%, *A*_420_ = 0.020) directly/indirectly validated covalent bonds (ester bond) between SA and CA. Adsorption kinetics revealed a two-stage process dominated by rapid surface adsorption (pseudo-first-order model, *R*^2^ = 0.982) followed by intraparticle diffusion, with chemisorption contributions emerging after 400 min (pseudo-second-order, after 400 min). Since the *n* value of the Sips model is close to 1, even though the *R*^2^ of the Freundlich model is high, the adsorption is mainly homogeneous and has a small amount of heterogeneity, according to Langmuir’s low *MRD*. The Temkin model and the thermodynamic data (Δ*H*° = −135.6 kJ/mol, Δ*S*° = −422.7 J/mol·K) show that the interaction is exothermic. Despite maintaining >85% efficiency over three regeneration cycles, significant capacity loss (~40%) in the fourth cycle suggested structural degradation, which decreased to about 16.7 mg/g after the fifth and sixth cycles, consistent with TGA results showing improved (thermal) stability. The adsorption mechanism may be dominated by electrostatic attraction (pH_pzc_ = 6.55) and hydrogen bonding. This work has a relatively high *q_e_* value compared to other past studies. The green synthesis of SA/CA gel material synthesized in this paper has the advantages of a wide source of raw materials, simple operation, high cost performance, green environmental protection, less pollution, good safety, etc. It can complete multiple dye removal under certain conditions and has a good development prospect.

## Figures and Tables

**Table 1:** Raw material *DG*% and Browning degree of SA/CA gel beads (0.5 g/L SA/CA beads, 300 mg/L, and 50 mL MB, pH = 7.0, T = 300 K).

Maillard Reaction Verification	0.66 mg/mL CA + 1 mg/mL SA	1 mg/mL CA + 1.5 mg/mL SA
*DG*%	11.40% ± 0.24%	13.90% ± 0.27%
Browning degree (*A*_420_)	0.033 ± 1.43 × 10^−4^	0.020 ± 2.11 × 10^−4^

**Table 2:** Names of SA/CA gel beads prepared with different reactant addition amounts and their *q_e_* values (0.5 g/L SA/CA beads, 300 mg/L, and 50 mL MB, pH = 7.0, T = 300 K).

SA/CA Mass Ratios	Amount of SA Solids Added (g)	Amount of CA Solids Added (g)	*q_e_*(mg/g)
SA/CA@0.75	1.00	0.75	277.52 ± 0.96
SA/CA@1		1.00	305.01 ± 0.22
SA/CA@1.25		1.25	275.57 ± 1.75
SA/CA@1.5		1.50	270.52 ± 7.98
SA/CA@1.75		1.75	258.63 ± 0.71

**Table 3:** Adsorption kinetic for MB adsorption onto SA/CA gel beads (0.5 g/L SA/CA beads, 300 mg/L, and 50 mL MB, pH = 7.0, T = 300 K).

Kinetic Models	Parameters	Values
Pseudo-first-order	*k_1_*	0.0022 ± 1.69 × 10^−4^
	*q_e, cal_*	309.25 ± 9.86
	*r* ^2^ *(COD)*	0.98
	*MRD*	10.19
Pseudo-second-order	*k_2_*	4.98 × 10^−6^ ± 9.26 × 10^−7^
	*q_e_*	396.15 ± 21.53
	*r* ^2^ *(COD)*	0.85
	*MRD*	11.93
Intra-particle diffusion	*k_id1_*	10.43 ± 0.90
	*C_1_*	−32.13 ± 13.76
	*r* ^2^ *(COD)*	0.93
	*k_id2_*	3.52 ± 1.60
	*C_2_*	154.41 ± 63.75
	*r* ^2^ *(COD)*	0.84

**Table 4:** Isotherm parameters for MB adsorption onto SA/CA gel beads (0.5 g/L SA/CA beads, different concentrations, and 50 mL MB, pH = 7.0, T = 300 K).

Isotherm Parameters								
*q_e,exp_* = 951 mg/g							*r* ^2^ *(COD)*	*MRD*
Langmuir	*q_m_*	1268.52 ± 135.64	*K_L_*	0.0015 ± 3.80 × 10^−4^			0.95	15.47
Freundlich	*K_F_*	28.58 ± 1.02	*n*	2.21 ± 0.0031			0.97	25.86
Temkin	*b*	10.27 ± 0.0060	*K_t_*	0.023 ± 4.40 × 10^−4^			0.97	17.45
Sips	*q_m_*	1270.46 ± 6.36	*β*_s_	0.0016 ± 2.23 × 10^−5^	*n*	0.85 ± 0.0060	0.99	18.48

**Table 5:** Thermodynamic parameters for MB adsorption onto SA/CA gel beads (0.5 g/L SA/CA beads, different concentrations and 50 mL MB, pH = 7.0, T = 290/300/310 K).

Thermodynamic Parameters	*q_e_*	ln*K_D_*	Δ*G*° (kJ/mol)	Δ*S*° (J/mol)	Δ*H*° (kJ/mol)	*r* ^2^ *(COD)*
290 K	1020.84	5.46 ± 0.081	−13.17 ± 0.20	−422.74 ± 5.31	−135.63± 1.59	0.99
300 K	951.14	3.48 ± 0.035	−8.68 ± 0.086			
310 K	935.47	1.90 ± 0.11	−4.90 ± 0.27			

## Data Availability

The original contributions presented in this study are included in the article. Further inquiries can be directed to the corresponding author.

## Abbreviations

The following abbreviations are used in this manuscript:

SA	Sodium alginate
CA	Casein
SA/CA	Sodium alginate-Casein compound gel material
OPA	o-Phthalaldehyde
SDS	Sodium dodecyl sulfate
MB	Methylene blue
SEM	Scanning electron microscope
FT-IR	Fourier transform infrared spectrometer
UV-light	Ultraviolet light
